# Extracellular vesicles in breast cancer metastasis: functional insights and advances in single-EV analysis

**DOI:** 10.1007/s10555-026-10382-7

**Published:** 2026-09-24

**Authors:** Yujin Lee, Yuling Wang, Peter Graham, Julia Beretov, Jie Ni

**Affiliations:** 1 St George and Sutherland Clinical Campuses, School of Clinical Medicine, UNSW Sydney, Kensington, NSW 2052 Australia; 2 Cancer Care Centre, St George Hospital, Kogarah, NSW 2217 Australia; 3 School of Natural Sciences, Faculty of Science and Engineering, Macquarie University, Sydney, NSW 2109 Australia

**Keywords:** Extracellular vesicles, Breast cancer, Metastasis, Single-EV analysis

## Abstract

Breast cancer (BC) metastasis remains a leading cause of mortality despite significant advances in screening and diagnosis. Extracellular vesicles (EVs), secreted by all living cells, carry biologically active cargo such as RNAs and proteins, playing a crucial role in cell-to-cell communication. Distinct EV subpopulations contribute to the tumour microenvironment, pre-metastatic niche formation, immune evasion, extracellular matrix remodelling, and ultimately promote BC organotropism. Beyond their biological roles, growing evidence highlights EVs as potential diagnostic and prognostic biomarkers, as well as viable therapeutic targets. However, the full scope of EV function in BC metastasis remains unclear, and their inherent heterogeneity and molecular complexity present considerable barriers to clinical translation. Addressing these challenges requires both a clear understanding of the distinct functions of each EV subpopulation and the application of advanced single-EV analysis techniques. In this review, we comprehensively examine the functional contributions of EV subpopulations to BC metastasis. Each RNA and protein cargo we discuss is assigned a graded evidence level. We further consider EV lipids, non-vesicular extracellular particles and current EV-directed therapeutic strategies. Finally, we highlight that single-EV analysis is a promising strategy to resolve this complexity and convert EV biology into clinically actionable biomarkers and targets.

## Introduction

The current clinical course of breast cancer (BC) reflects a paradox: although treatment of localised disease has led to significantly improved survival rates, the progression of cancer to distant metastases continues to present a major clinical challenge. Following initial treatment, approximately 20–30% of patients with early-stage BC eventually develop distant metastases within 10 years, and metastasis is responsible for nearly 90% of BC-related deaths [1].

Cancer metastasis involves sequential stages, involving the detachment and spread of cancer cells from the primary tumour, cells entering the bloodstream or lymphatic system, which then extravasate to distant organs, and proliferate within the new environment, as outlined in a previous review [2]. However, when studying a single BC cell population to investigate metastasis, BC cells exhibit diverse characteristics due to genetic variations and interactions with other cells or the extracellular matrix, leading to different metastatic phenotypes [3, 4]. These complexities present challenges in BC therapeutic research and in predicting drug responses and resistance to therapies. For this reason, BC researchers have shifted their focus to the tumour microenvironment (TME) [5]. The TME plays a crucial role in tumour initiation, progression, migration and invasion, as reviewed in [6]. Within this milieu, in recent years, extracellular vesicles (EVs), released by cells, have gained significant attention, influencing various hallmarks of cancer. In BC, different EV subpopulations, mainly small EVs (sEVs), large EVs (lEVs), and apoptotic bodies, play distinct roles in promoting tumour progression and metastasis.

EVs, membrane-bound particles, are lipid-bilayered vesicles secreted by diverse cell types that carry proteins, lipids, DNA, and RNA that mediate intercellular communication by transporting bioactive cargo between cells [7]. EVs represent a heterogeneous group of membrane-bound vesicles that are classified into several subpopulations based on their size and biological characteristics. Typically, sEVs or exosomes measure from 50 to 200 nm in diameter, whereas lEVs are 150 nm to 500 nm and apoptotic bodies range from 500 to 4000 nm [8]. Following the MISEV guidelines, we use the terms sEVs and lEVs as practical size-based categories rather than as definitive indicators of vesicle origin. Studies were grouped into the sEV or lEV sections according to the terminology and particle sizes reported in the original publications, supported where possible by the available isolation and characterisation data. However, because EV size ranges overlap and commonly used isolation methods can also recover non-vesicular extracellular particles, such as exomeres and supermeres, we do not use sEV and lEV interchangeably with exosome and microvesicle, respectively.

EVs reflect the molecular composition of their cell of origin and are abundant in human body fluids, serving as important messengers contributing to key features of cancers such as progression, metastasis, and angiogenesis [9]. In particular, as a key mediator of intercellular communication among the EV subpopulations, lEVs and sEVs play important roles in delivering bioactive cargo associated with cancer-related processes (progression, metastasis, and angiogenesis). Studies have shown that sEVs in distant target organs facilitate integrin uptake by cells from the organ. This, in turn, establishes the microenvironment required to enable the initial metastasis via seeding tumour cells [10]. Similarly, lEVs transport surface antigens, oncogenic products, and adhesion molecules on their surface or within vesicles and deliver them to distant sites, playing a crucial role in the invasion and metastasis of cancer [11, 12].

**Fig. 1:**
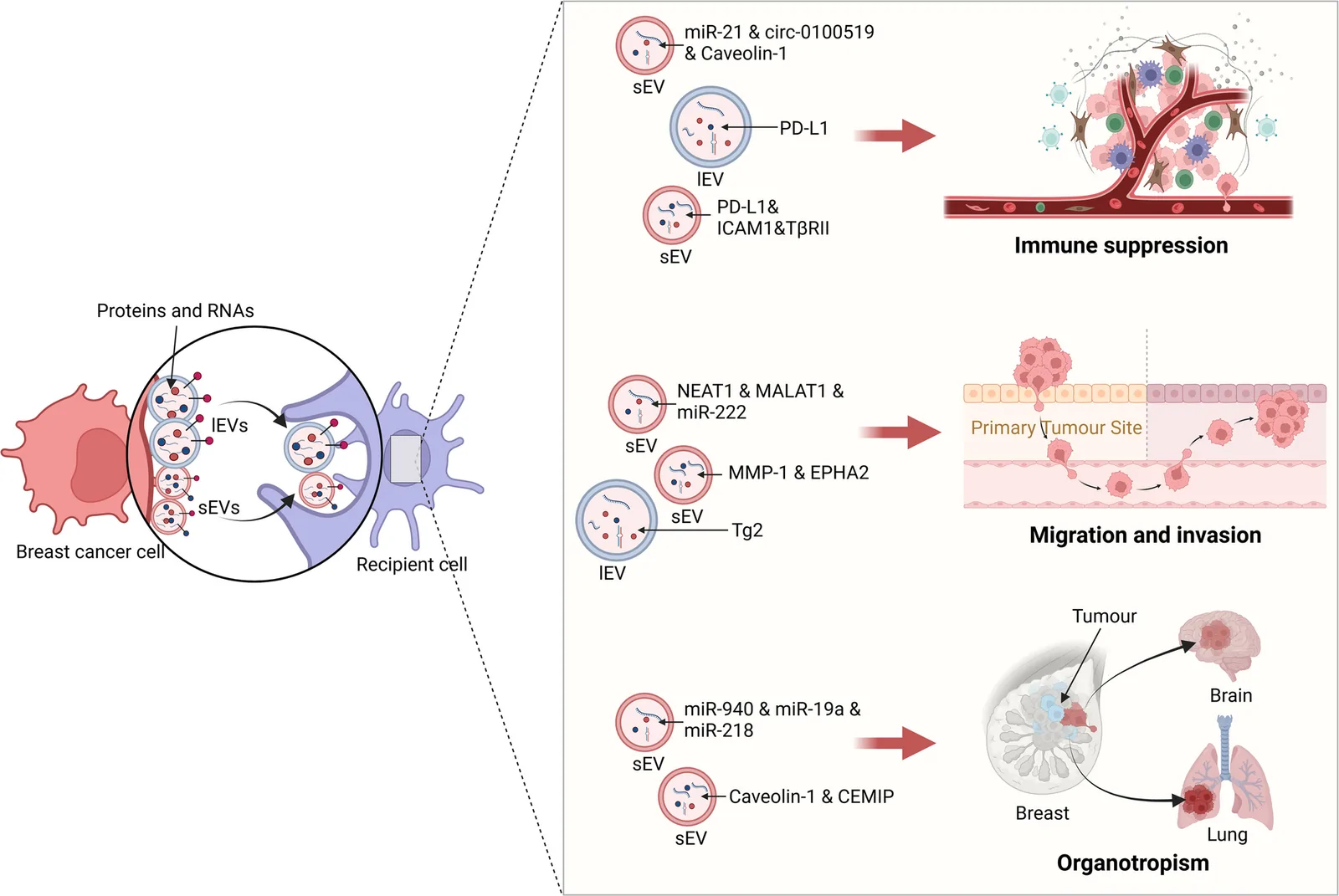
Roles of EV subpopulations in breast cancer metastasis. Breast cancer cells release sEVs and lEVs carrying various RNAs and proteins and deliver them to neighbouring cells. This delivery remodels the tumour microenvironment by inducing immune suppression, migration and invasion, thereby promoting pre-metastatic niche formation. Furthermore, miRNAs and proteins within EVs are also associated with organotropic niche conditioning and distant colonisation. Created in BioRender. BioRender.com/05qf578

Despite significant research efforts, the clinical translation of EVs remains challenging, with the Phase I clinical trials success rate for oncology drug development remaining only around 3.4% [13]. The gap between experimental findings and real-world outcomes partly stems from the difficulty in replicating the complexity of cancer biology in current functional studies and partly from the intrinsic heterogeneity of EV populations. To overcome these challenges, this review discusses how lEV and sEV cargoes, including RNAs and proteins, contribute to distinct stages of BC metastasis (Fig. 1). Importantly, each EV RNA and protein cargo discussed is accompanied by a graded evidence level. To our knowledge, this is the first review to apply such evidence grading systematically to EV cargoes in BC metastasis, and it is intended to allow readers to weigh candidate cargoes according to the strength of the data underpinning them. We further highlight emerging single-EV analysis approaches as tools to resolve vesicle heterogeneity and to identify EV subpopulations specific to metastasis that may be obscured in conventional bulk EV studies. Techniques such as nano-flow cytometry, droplet-based assays, Raman spectrometry-based approaches, aptamer-based detection, and microscopy-based single-EV imaging now allow EV phenotypes to be assessed at the individual vesicle level. These approaches may enable the linkage of individual EVs to metastatic functions, supporting the development of clinically useful EV-based biomarkers and therapeutic targets.

## The function of EV subpopulations in BC metastasis

EVs play a crucial role in mediating intercellular communication within the BC microenvironment, particularly through interaction between tumour cells and stromal cells. EVs protect their RNA cargo from degradation in the extracellular environment and facilitate intercellular communication by transferring RNAs between cells. The pro-metastatic functions of EVs are driven by their diverse cargo, which includes proteins and nucleic acids. These molecules are internalised by recipient cells, where they modify the recipient cells’ phenotype and modulate the metastatic cascade [14]. MicroRNAs (miRNAs) are small non-coding RNAs (ncRNAs), typically 18–24 nucleotides in length, that bind to target mRNAs to suppress their translation into proteins [15]. Similarly, long non-coding RNAs (lncRNAs) are highly conserved RNA molecules longer than 200 nucleotides that do not encode proteins [16]. lncRNAs regulate gene expression by “sponging” miRNAs, a mechanism known as the competing endogenous RNA (ceRNA) mechanism [17]. Circular RNAs (circRNAs), a class of ncRNAs characterised by a covalently closed loop structure lacking 5′ caps and 3′ poly(A) tails, are generated from protein-coding genes via back splicing and can act similarly to miRNAs and other ncRNAs, and may also serve as templates for cap-independent translation, as reviewed in [18]. Owing to their circular and more stable structure along with being highly resistant to exonuclease-mediated degradation, circRNAs are highly stable and are often enriched in extracellular vesicles including exosomes and microvesicles [19]. Together, they constitute a coordinated network that can be transferred to recipient cells to reprogramme post-transcriptional gene expression and facilitate pro-metastatic signalling [20]. In addition to RNA cargo, EVs also carry metastasis-associated proteins. Proteomic analysis of sEV proteins derived from BC cell lines has identified numerous metastasis-associated proteins within sEVs, including Ezrin, annexin A5, annexin A6, tenascin, and extracellular matrix metalloproteinase inducer (EMMPRIN/CD147) [21]. In this section, we will explore the detailed functions of these cargoes in modulating BC metastasis.

### sEV RNAs function in BC metastasis

Common BC sEV RNAs promote metastasis through several convergent mechanisms, including increased endothelial permeability, induction of epithelial-mesenchymal transition (EMT), immune modulation, and pre-metastatic niche (PMN) formation. As described in a previous review, prior to overt metastasis, interactions between primary tumour-derived factors and stromal components alter the microenvironment to establish a PMN [22]. This niche is characterised by several coordinated changes, including immunosuppression, inflammation, angiogenesis, and cellular reprogramming [23]. In particular, BC-derived sEV ncRNAs can reprogramme macrophages towards tumour-associated phenotypes, creating an immunosuppressive microenvironment that facilitates PMN formation and subsequent metastatic progression.

Several BC-derived sEV RNAs contribute to macrophage reprogramming during PMN formation. For example, sEV miR-33, miR-130, and circ-0100519 contributed to activating macrophages into tumour-associated macrophages (TAMs), which were highly associated with BC migration and invasion [24, 25]. Mechanistically, sEV circ-0100519 promoted ubiquitin-specific peptidase 7 (USP7)-mediated deubiquitylation and stabilisation of nuclear factor erythroid 2-related factor 2 (NRF2), a transcription factor involved in macrophage polarisation, thereby inducing TAM-like phenotype [25]. Additionally, sEV miR-138-5p promoted TAM-like polarisation by suppressing epigenetic factor lysine demethylase 6B (KDM6B), thereby facilitating triple-negative breast cancer (TNBC) lung metastasis [26]. Separately, Ras-related protein Rab-5a (RAB5A) in TNBC cells enhanced sEV secretion and the transfer of sEV miR-21 to macrophages, where sEV miR-21 suppressed Pellino-1 (PELI1) and promoted TAM-like polarisation [27]. sEV miR-21 also suppressed programmed cell death 4 (PDCD4) in osteoclast precursors, thereby promoting BC osteoclastogenesis and supporting the formation of a bone metastatic niche [28]. Such immune and stromal reprogramming can further promote immune escape in BC. In TNBC and ER^+^PR^+^ BC models, endoplasmic reticulum stress increases the release of sEV miR-27a-3p from BC cells. In macrophages, sEV miR-27a-3p suppresses membrane-associated guanylate kinase, WW and PDZ domain-containing protein 2 (MAGI2), a scaffold protein that helps maintain phosphatase and tensin homolog (PTEN) stability [29]. Loss of MAGI2 weakens PTEN-mediated inhibition of phosphoinositide 3-kinase (PI3K)/AKT signalling, leading to increased programmed death-ligand 1 (PD-L1) expression. These changes reduce cytokine secretion and promote T cell apoptosis, thereby enabling immune evasion in BC. Moreover, sEV circ-EGFR promoted autophagy in recipient TNBC cells through two mechanisms [30]. First, it caused Annexin A2 to relocate to the plasma membrane, allowing transcription factor EB (TFEB) to enter the nucleus and activate autophagy-related genes. Second, circ-EGFR suppressed miR-224-5p expression, which increased the expression of the autophagy regulators in TNBC cells. Therefore, autophagy facilitated by sEV circ-EGFR might be a crucial factor in the high metastatic and invasive features of TNBC. sEV circ-PSMA1 acted as a sponge for miR-637, preventing miR-637 from suppressing AKT1 [20]. The resulting activation of AKT1/β-catenin signalling promoted immune suppression and enhanced TNBC metastasis.

sEV RNAs in BC also mediate bidirectional communication between tumour cells and fibroblasts. It has also been reported that sEV miR-125b and miR-370-3p from TNBC cells were delivered to fibroblasts, facilitating their differentiation into cancer-associated fibroblasts (CAFs) and stimulating pro-inflammatory factor production [31, 32]. In turn, sEV miR-500a-5p and miR-18b, derived from CAFs, were transferred back to TNBC and ER^+^PR^+^ BC cell lines, resulting in downregulating ubiquitin-specific peptidase 28 (USP28) and transcription elongation factor A like 7 (TCEAL7), thereby enhancing metastatic features [33, 34]. Interestingly, sEV miR-370-3p from TNBC cells also downregulated cylindromatosis, another member of the USP family [32]. Together, these findings support reciprocal sEV-mediated communication between TNBC cells and CAFs rather than a single experimentally established signalling loop. TNBC-derived miR-370-3p activates CAFs through suppression of cylindromatosis lysine 63 deubiquitinase (CYLD) and subsequent activation of NF-κB signalling, whereas CAF-derived miR-500a-5p enhances metastasis phenotypes in BC cells by suppressing USP28 and TCEAL7. This process facilitated inflammatory signalling and activated NF-κB signalling [35, 36], which is well-known for its role in BC growth and TNBC migration [37].

In addition to immune and stromal reprogramming, several sEV RNAs also contribute to angiogenesis and vascular remodelling within the PMN. Notably, sEV miR-105, secreted by highly metastatic TNBC cells, inhibited the tight junction protein zonula occludens-1 (ZO-1), thereby disrupting the endothelial barrier [38]. This disruption increases vascular permeability and creates a permissive microenvironment that facilitates the early dissemination of tumour cells to distant organs in BC.

Whilst previous findings describe the changes during PMN formation, distinct sets of sEV RNAs actively contribute to the metastatic cascade once dissemination has begun. Highly metastatic TNBC cells significantly expressed sEV miR-10b and miR-222 compared to low metastatic BC cells [39, 40]. The sEV miR-222 promoted BC cell invasion and migration by suppressing PDZ and LIM domain protein 2 (PDLIM2), because PDLIM2, a tumour suppressor that normally limits the NF-κB signal pathway, is lost, resulting in sustained NF-κB signal pathway [40]. sEV miR-10b in highly metastatic TNBC cells promoted the invasive behaviour of recipient cells by suppressing homeobox D10 (HOXD10), a transcription factor that normally limits the expression of genes involved in cell migration and invasion [39]. However, the downstream mechanisms responsible for this effect remain unclear. One lncRNA, nuclear paraspeckle assembly transcript 1 (NEAT1), is highly expressed in the serum of BC patients. Both *in vitro* (TNBC and ER + PR + BC cell lines) and *in vivo*, NEAT1 functions as a sponge for miR-141-3p, leading to the upregulation of Kruppel‐like factor 12 (KLF12) and, consequently, enhanced invasion and migration [41]. Another lncRNA, metastasis-associated lung adenocarcinoma transcript 1 (MALAT1) within sEVs promoted vasodilator-stimulated phosphoprotein (VASP) expression by inhibiting miR-1-3p in ER + PR + BC cell lines, thereby facilitating metastasis [42]. In addition, sEV miR-7641 levels were markedly higher in metastatic BC cell lines and in the plasma of BC patients with distant metastasis [43]. Furthermore, sEV miR-361-3p targeted the transcriptional regulators ETS variant transcription factor 7 (ETV7) and basic leucine zipper ATF-like transcription factor 2 (BATF2). Their suppression was associated with increased plasminogen activator inhibitor-1 (PAI-1) expression and extracellular signal-regulated kinase (ERK) activation, which promoted BC cell survival and migration [44–46]. Consistently, in BC patients’ plasma, sEV miR-361-3p was positively correlated with malignancy [46].

In addition to these general pro-metastatic effects, several sEV RNAs also contribute to distant spread and, in some cases, organ-specific metastasis. Related to lung tropism, sEV miR-200 promotes metastatic colonisation by enhancing the metastatic capacity of recipient BC cells [47]. In liver metastasis, sEV miR-4443 inhibited tissue inhibitors of metalloproteinase 2 (TIMP2) and upregulated matrix metalloproteinases (MMPs) in recipient cells, facilitating metastatic progression to the liver [48].

BC brain metastasis is also linked to sEV-mediated crosstalk within the brain microenvironment. The brain metastatic BC cells secreted a high level of sEV miR-1246 and miR-1290, which activated astrocytes in the brain metastatic niche [49]. Separately, sEV miR-1246 derived from brain-metastatic BC cells promoted angiogenesis in the brain metastatic microenvironment, potentially contributing to brain metastasis of BC [50]. In addition, brain-metastatic TNBC-derived sEVs transferred miR-301a-3p and lncRNA GS1-600G8.5 to astrocytes, leading to blood–brain barrier disruption and thereby facilitating brain metastasis of BC [51, 52]. In turn, astrocytes can also deliver miR-19a via sEVs to brain-metastatic BC cells, downregulating PTEN expression levels [53]. This downregulation increased cytokine chemokine ligand 2 (CCL2) expression, resulting in brain metastasis of BC. Beyond these signalling changes, sEV cargo can further remodel the brain metastatic niche through metabolic mechanisms. sEV miR-199b-5p was more highly expressed in BC patients with brain metastasis than in those with metastases to other organs [54]. This miRNA inhibited solute carrier transporters required for neuron coupling, impairing uptake of glutamate, glutamine, and lactate [54]. Such metabolic disruption may increase the availability of these substrates in the microenvironment, thereby supporting brain metastatic growth.

In terms of the bone metastasis of BC, bone metastatic TNBC-derived sEVs transferred miR-940 to mesenchymal stem cells and facilitated the osteogenic differentiation of the mesenchymal stem cells, establishing the bone-metastatic microenvironment in BC [55]. Also, sEV miR-19a and Integrin-Binding Sialoprotein (IBSP) were significantly upregulated in the serum of bone metastatic ER^+^ BC patients and bone trophic ER^+^ BC cell lines [56]. The study suggested that sEV IBSP derived from BC cell lines recruits bone stromal cells, and sEV miR-19a enhances their differentiation, which could be an initial bone-metastatic factor in ER^+^ BC. Additionally, miR-218 was implicated in bone-metastatic BC via downregulating type I collagen and suppressing procollagen processing in bone stromal cells [57].

A detailed summary of EV RNA functions in BC metastasis is provided in Table 1. Through these mechanisms, sEV ncRNAs ultimately facilitate highly metastatic behaviour and organ-selective dissemination through interconnected regulatory networks rather than isolated pathways. Therefore, a more integrated and system-level understanding of metastasis is needed, particularly regarding the relationships between the ncRNA and protein compositions of sEVs. Their biological effects may also depend on coordination with sEV-associated proteins. Therefore, understanding the roles of these sEV proteins is necessary to define how sEVs facilitate metastasis in BC. Another caveat when interpreting Table 1 is that the overexpression of a miRNA in donor cells increased its abundance in the conditioned medium as a whole, and not solely within vesicles [58]. The phenotype observed in recipient cells could be attributed to non-EV carriers including extracellular particles (EPs, further discussed later), and extracellular ribonucleoprotein complexes, especially for low-abundance species, since a fractional average copy number per vesicle requires either a highly enriched vesicle subpopulation or an alternative carrier to account for a dose-dependent effect [58]. We therefore regard the RNA cargoes in Table 1 as having robust implications as mediators of BC metastasis, and their vesicular delivery is considered plausible, but in most cases, not formally demonstrated.

**Table 1:** EV RNAs function in BC metastasis

Name	EV subtype	BC subtype	EV-associated effects on BC metastasis	Evidence level	Reference
Circ-0100519	sEV	TNBC	Deubiquitinate NRF2 Activate TAMs	3	[25]
Circ-EGFR	sEV	TNBC	Inhibit miR-224-5p Induce high level of autophagy	2	[30]
Circ-PSMA1	sEV	TNBC	Inhibit miR-637 Related to immune suppression in BC	2	[20]
GS1-600G8.5	sEV	TNBC	Contribute to blood–brain barrier disruption during metastasis	2	[52]
MALAT1	sEV	ER^+^PR^+^ BC	Upregulate VASP by targeting miR-1-3p	1b	[42]
miR-10b	sEV	TNBC	Inhibit HOXD10	2	[39]
miR-105	sEV	TNBC	Inhibit a tight junction protein ZO-1 to increase vascular permeability	1b	[38]
miR-1246 & miR-1290	sEV	TNBC	Related to highly metastatic BC Promote brain metastasis of BC	1b	[49, 50]
miR-125b	sEV	TNBC	Activate CAFs Stimulate pro-inflammatory factors	1b	[31]
miR-130 & miR-33	sEV	TNBC	Activate TAMs	2	[24]
miR-138-5p	sEV	TNBC	Inhibit KDM6B Activate TAMs Related to BC lung metastasis	2	[26]
miR-18b	sEV	TNBC ER^+^PR^+^ BC	Downregulate TCEAL7 for activating PMN	2	[34]
miR-19a	sEV	TNBC ER^+^ BC	Related to brain metastasis of BC Downregulate PTEN and upregulate CCL2 Facilitate differentiation of the bone stromal cells	3	[53, 56]
miR-199b-5p	sEV	TNBC	Related to brain metastasis of BC Impair metabolism by inhibiting solute carrier transporters	2	[54]
miR-200	sEV	HER2^+^ BC TNBC	Promote metastasis of otherwise weakly metastatic cells either nearby or at distant sites and conferred to these cells the ability to colonize distant tissue	1b	[47]
miR-21	sEV	TNBC	Inhibit PELI1, RAB5A, and PDCD4 Activate TAMs Related to BC bone metastasis	2	[27, 28]
miR-218	sEV	TNBC	Downregulate type I collagen to interrupt procollagen processing of bone stromal cells	2	[57]
miR-222	sEV	TNBC	Target tumour suppressor gene and activate NF-κB signal pathway	2	[40]
miR-27a-3p	sEV	TNBC ER^+^PR^+^ BC	Inhibit MAGI2 Upregulate PD-L1 Related to immune escape in BC	1b	[29]
miR-301a-3p	sEV	TNBC	Interact with astrocytes and promote brain metastasis	3	[51]
miR-361-3p	sEV	HER2 + BC TNBC	Upregulate PAI-1/ERK signalling Related to highly metastatic BC	1b	[46]
miR-370-3p	sEV	TNBC	Downregulate USP family Activate CAFs	1b	[32]
miR-4443	sEV	TNBC	Related to liver metastasis of BC Related to highly metastatic BC Inhibit TIMP2 Upregulate MMPs	1b	[48]
miR-500a-5p	sEV	TNBC ER^+^PR^+^ BC	Downregulate USP28 Facilitate inflammatory signalling Activate NF-κB pathway	1b	[33]
miR-7641	sEV	TNBC	Related to highly metastatic BC	2	[43]
miR-940	sEV	TNBC	Enhance osteogenic differentiation of the mesenchymal stem cells Related to bone metastasis of BC	2	[55]
NEAT1	sEV	TNBC ER^+^PR^+^ BC	Upregulate KLF12 by targeting miR-141-3p	1b	[41]

Evidence level: 1b bidirectional cargo manipulation with *in vivo* confirmation without EV route-dependency established, 2 single-direction cargo manipulation with recipient-cell functional studies, 3 transfer/uptake with an associated phenotype with no cargo-specific manipulation

### sEV proteins function in BC metastasis

sEV proteins contribute to BC metastasis by promoting invasion, angiogenesis, immune evasion, and pre-metastatic niche formation, highlighting their importance in tumour progression and potential clinical applications. In TNBC, integrins αv and β1 co-localised with CD63 within sEVs [59]. The sEV integrins αvβ1 complex helped these sEVs bind to fibronectin in the extracellular matrix. This increased their local retention and uptake by fibroblasts. After uptake by fibroblasts, sEV Survivin increased superoxide dismutase 1 (SOD1) expression. This promoted the development of a CAF-like phenotype and supported PMN formation in BC [60]. Similarly, ER^+^PR^+^ BC cells released sEVs containing mutated p53 proteins with a gain-of-function phenotype bound to heat shock protein 90 (HSP90) [61]. HSP90 stabilised mutant p53 and supported its transfer to fibroblasts. The transferred proteins activated CAFs and helped establish a pro-metastatic microenvironment. In sEVs derived from TNBC cells, signal-induced proliferation-associated 1 (SIPA1) was highly upregulated [62]. Increased sEV SIPA1 was associated with greater macrophage infiltration and migration. PD-L1, an immune checkpoint ligand, was highly expressed on sEVs derived from BC patients’ plasma [63]. sEV PD-L1 directly bound to the programmed cell death receptor 1 (PD-1) on T cells and inhibited their activity, allowing TNBC cells to escape immune surveillance [64]. Another study found that sEV PD-L1 was more abundant in TAMs than in BC cells [65]. These vesicles promoted M2-like macrophage polarisation and created an immunosuppressive environment that supported invasion and metastasis. Beyond PD-L1, ubiquitin-specific peptidase 8 (USP8) also contributed to immune evasion in metastatic BC [66]. USP8 increased the loading of TGF-β type II receptor (TβRII) into BC cell-derived sEVs and helped its transfer to CD8^+^ T cells. In these cells, sEV TβRII activated SMAD family member 3 (SMAD3), which worked with T-cell factor 1 to promote a T-cell exhaustion phenotype [66, 67]. TβRII-containing sEVs also increased cancer stemness and metastasis in a BC mouse model. sEV-associated intercellular adhesion molecule 1 (ICAM1) was also linked to CD8^+^ T-cell exhaustion, TNBC tumour growth, and bone metastasis [68]. However, the signalling events that connect ICAM1 to T-cell exhaustion remain unclear. Furthermore, highly enriched thrombospondin-1 (TSP1) in sEVs derived from TNBC cells downregulated intercellular junction proteins such as vascular endothelial cadherin and ZO-1. As these proteins help maintain endothelial junctions, their downregulation weakened the vascular barrier and facilitated the migration and invasion of TNBC [69]. Similarly, TNBC-derived sEVs containing nucleoside diphosphate kinase (NDPK) also increased vascular permeability [70]. By weakening the endothelial barrier, these proteins may help circulating BC cells enter distant tissues and establish PMNs.

In the context of BC pro-metastasis, several proteins within sEVs have been identified as being highly expressed and associated with TNBC cell adhesion, invasion, and metastasis. These include L-plastin, an actin-binding protein involved in cell movement [71] and EGF-like repeats and discoidin domains 3 (EDIL3), an extracellular matrix protein that supports cell adhesion [72]. Interestingly, sEV glycosylation has also been implicated in BC metastasis. The glycosylation of sEV integrin β1 has been linked to BC metastasis. Low levels of bisecting GlcNAc modification on sEV integrin β1 increased its pro-metastatic activity in BC cell lines [73]. EPH receptor A2 (EPHA2) carried by TNBC-derived sEVs has been implicated in metastasis through distinct mechanisms. One study showed that sEV EPHA2 disrupted the endothelial barrier and increased vascular permeability, potentially facilitating PMN formation [74]. In a separate study, EPHA2 was highly expressed in sEVs from highly metastatic TNBC cells and promoted angiogenesis through activation of AMP-activated protein kinase (AMPK) signalling [75]. These findings indicate that sEV EPHA2 may support BC metastasis through both vascular barrier disruption and pro-angiogenic signalling. Matrix metalloproteinase-1 (MMP-1) was highly expressed in sEVs derived from highly metastatic TNBC cell lines [76]. Notably, the level of sEV MMP-1 was elevated in the serum of BC patients with distant metastasis compared to those without metastasis. This finding suggested that sEV MMP-1 may promote BC metastasis by activating protease-activated receptor 1 (PAR1), a G protein-coupled receptor involved in cell migration and invasion.

Furthermore, organotropism is also found to be controlled by distinct sEV proteomic signatures. Using proteomic analyses, Hoshino et al. demonstrated that specific sEV integrins on BC-derived sEVs mediated organ-specific interactions during metastasis [10]. Once taken up by resident cells in the lung or liver, these sEVs activated Src phosphorylation and induced pro-inflammatory gene expression in the lung and liver. These changes helped establish a tissue environment that supported BC metastatic colonisation. Similarly, transforming growth factor beta 1 (TGFβ1) carried by BC-derived sEVs increased fibronectin expression in liver endothelial cells, promoting tumour cell adhesion and helping establish a metastatic niche in the liver [77]. sEV Caveolin-1 also contributed to inflammatory priming before BC lung metastasis. sEV Caveolin-1 regulated integrin α6β4 and toll-like receptor 4 (TLR4) signalling, and further promoted cytokine release, TAM activation, and angiogenesis in BC lung metastasis [78, 79]. Likewise, BC-derived sEV Caveolin-1 promoted extracellular matrix remodelling in lung fibroblasts, increased the expression of PMN-associated genes and inflammatory chemokines in lung epithelial cells, and supported M2-like polarisation of lung macrophages [80]. In the context of BC brain metastasis, Busatto et al. suggested that the EVs derived from brain metastatic BC cells may cross the blood–brain barrier via transcytosis rather than by disrupting barrier integrity [81]. This process may involve the upregulation of RAB11 family-interacting proteins 3 and 5 (Rab11fip3 and Rab11fip5), two proteins that are associated with structural stability and transport across endothelial cells. Meanwhile, sEV cell migration-inducing and hyaluronan-binding protein (CEMIP) was found to be selectively upregulated in brain-metastatic BC cells, but not in other metastatic cells [82]. sEV CEMIP created a niche that supported BC brain metastasis by enhancing the expression of inflammatory cytokines related to vascular remodelling [82]. A detailed list of EV protein functions in BC metastasis is shown in Table 2**.**

**Table 2:** EV proteins function in BC metastasis

Name	EV subtype	BC subtype	Function in BC metastasis	Evidence level	Reference
Caveolin-1	sEV	TNBC ER^+^ BC	Related to lung metastasis of BC Regulate inflammatory priming pathways and PMN marker expression Activate TAM Remodel extracellular matrix in lung fibroblasts	1b	[78–80]
CD44	lEV	TNBC	Reduce focal adhesions and actin cytoskeleton rearrangement	2	[83]
CEMIP	sEV	TNBC	Related to vascular remodelling and brain metastasis of BC Enhances pro-inflammatory vascular niche	1b	[82]
EDIL3	sEV	TNBC	Promote BC invasion through integrin-FAK signalling pathway	1b	[72]
EPHA2	sEV	TNBC	Inhibit tight junction proteins to increase PMN for organotropism Activate AMPK signalling related to pro-angiogenesis and metastasis	1b	[74, 75]
GOF p53	sEV	ER^+^PR^+^ BC	Bind to HSP90 and then activate CAFs	2	[61]
ICAM1	sEV	TNBC	Induce exhaustion of CD8^+^ T cells	2	[68]
Integrins	sEV	TNBC	Prepare PMN for organotropism via activation of Src phosphorylation and pro-inflammatory gene expression	2	[10]
Integrins αvβ1	sEV	TNBC	Enhance EV uptake by fibroblasts	2	[59]
Integrin β1	sEV	ER^+^PR^+^ BC	Bisecting GlcNAc modification of sEV Integrin β1 modulates migration and metastasis	2	[73]
L-plastin	sEV	TNBC	Mediate osteoclast activation by human breast cancer cells	1a	[71]
MMP-1	sEV	TNBC	Interact with G protein receptor protease-activated receptor 1 to facilitate metastasis	3	[76]
NDPK	sEV	TNBC	Induce pulmonary vascular leakage and cell migration for BC PMN construction in lung	2	[70]
PD-L1	sEV/lEV	TNBC	Bind to PD-1 to inhibit T cell killing activity Promote M2-phenotype macrophage polarisation Contributes to irradiation-induced immune evasion	2	[64, 84, 85]
SIPA1	sEV	TNBC	Enhance macrophage infiltration Initiate PMN	2	[62]
Survivin	sEV	TNBC	Activate CAFs and promote PMN	1b	[60]
Tg2	lEV	TNBC	Activate fibroblasts Promotes tumour stiffening and metastasis	3	[86]
TGF-β1	sEV	TNBC	Promoted BC cell adhesion Related to BC liver metastasis	3	[77]
TSP1	sEV	TNBC	Downregulate intercellular junction proteins to enhance migration and invasion	1b	[69]
TβRII	sEV	TNBC	Transferred by sEVs to CD8^+^ T cells Induce exhaustion of CD8^+^ T cells Enhance stemness and metastasis	2	[67]

Evidence level: 1a EV-route dependency established with bidirectional cargo manipulation and *in vivo* confirmation, 1b bidirectional cargo manipulation with *in vivo* confirmation without EV route-dependency established, 2 single-direction cargo manipulation with recipient-cell functional studies, 3 transfer/uptake with an associated phenotype with no cargo-specific manipulation

### lEV function in BC metastasis

In BC, lEVs contribute to tumour progression and metastasis by transferring bioactive molecules such as proteins, lipids, DNA, and RNA to recipient cells. Although lEVs have been studied less extensively than sEVs, emerging evidence now positions lEVs as critical mediators of the metastatic cascade with distinct biological properties and molecular repertoires [87]. lEVs can promote metastasis through both immune and non-immune mechanisms. PD-L1-positive lEVs isolated from patients with TNBC suppressed cytotoxic T-cell activity and promoted macrophage polarisation towards an M2-like phenotype. In macrophages, this effect was linked to activation of the TANK-binding kinase 1 (TBK1)/signal transducer and activator of transcription 6 (STAT6) pathway, which supports M2-like polarisation and an immunosuppressive microenvironment [84]. Similarly, irradiation induced the release of PD-L1-containing lEVs that suppressed cytotoxic T-cell activity in a partly PD-L1-dependent manner [85]. Beyond immune modulation, lEVs from highly metastatic or chemotherapy-exposed BC cells enhanced metastatic potential in low-metastatic BC cells by reducing focal adhesions and inducing actin cytoskeleton rearrangement, mediated by CD44 within lEVs [83].

lEVs also remodel non-malignant components of the tumour microenvironment. Transglutaminase 2 (Tg2)-enriched lEVs released by weakly migratory metastatic BC cells activated fibroblasts and increased tumour stiffness, resulting in tumour-cell dissemination [86]. In TNBC, neoadjuvant chemotherapy increased the release of phosphatidylserine-exposing lEVs. These lEVs promoted platelet activation and coagulation, increased endothelial permeability and facilitated tumour-cell transendothelial migration [88]. Collectively, these findings indicate that lEVs support BC metastasis by immune evasion, altering tumour-cell behaviour, and remodelling stromal and vascular components of the metastatic environment.

As discussed above, the biological effects of EVs during BC metastasis are likely to reflect the combined influence of RNA and protein cargoes rather than the actions of individual molecules independently. Different EV subpopulations may produce overlapping biological effects by carrying similar molecules, while subtype-specific cargoes may enable them to perform distinct roles at different stages of metastasis. Comparative proteomic profiling in BC cell lines supports this possibility. In TNBC cells, lEVs and sEVs contained a substantial number of overlapping proteins, although several proteins, including functional metabolic enzymes, were specifically enriched in lEVs [89]. Further proteomic analyses revealed that protein profiles varied according to both EV subpopulation and BC cell line, highlighting the molecular heterogeneity of EVs [90]. These shared and distinct cargo profiles may influence how different EV subpopulations regulate tumour-cell behaviour and the metastatic microenvironment.

Beyond RNA and protein cargo, differences between these EV subpopulations may also extend to lipid composition. One study showed that adipocyte-derived sEVs were enriched in cholesterol, whereas lEVs contained higher levels of externalised phosphatidylserine [91]. Another study reported that phosphatidylserine on EV membranes may contribute to macrophage recognition and EV uptake [92]. Therefore, subtype-specific lipid profiles may play an important role in BC progression and provide additional biomarkers for BC detection and classification [93, 94]. However, further studies are required to determine how lipids are distributed among EV subpopulations and whether these differences directly affect BC progression and metastasis.

In BC, both sEVs and lEVs have been implicated in metastatic progression, although their reported contributions may differ in emphasis. sEVs appear to be more associated with organotropism and pre-metastatic niche conditioning. This may be attributed to their endosomal biogenesis and small size, which may support selective enrichment of tissue-tropic surface molecules such as integrins and stable circulation and uptake at distant sites. In contrast, the involvement of lEVs appears to become more evident under therapy-induced stress. Cytotoxic exposure can promote plasma membrane shedding and increase lEV release, enabling the packaging and transfer of larger or more complex cargo [95, 96]. The key differences between sEVs and lEVs in terms of biogenesis, cargo, uptake, organotropism, and biomarker potential are summarised in Table 3.

**Table 3:** Comparison of sEVs and lEVs in BC

Features	sEVs	lEVs
Biogenesis	Endosomal pathway and multivesicular bodies	Outward budding from the plasma membrane
Major cargoes reported in BC	Extensively characterised RNAs and proteins, including miRNAs, circRNAs, lncRNAs, integrins, PD-L1, and CEMIP. Less characterised lipids	Proteins, lipids, DNA, and RNAs, including PD-L1 and CD44; fewer characterised cargoes
Major metastatic functions	Promote endothelial permeability, EMT, immune evasion, stromal and macrophage reprogramming, angiogenesis, and PMN formation	Immune suppression, cytoskeletal rearrangement, loss of focal adhesion, platelet and fibroblast activation, tumour stiffening, and therapy-induced metastatic progression
Uptake and function	Uptake by tumour endothelial, immune and stromal cells	Functional transfer to tumour, immune, and stromal cells has been demonstrated, but the uptake mechanisms remain unclear
Organotropism	Associated with lung, liver, brain, and bone metastasis	Organ-specific targeting remains unclear
Biomarker potential	Multiple circulating RNAs and proteins evaluated in clinical samples	Emerging potential, but limited clinical evidence

Importantly, findings should be interpreted in the context of the broader lEV population, as current isolation methods lack sufficient resolution to reliably distinguish each EV subtype. A novel EV subtype termed migrasomes, an important mediator of intercellular communication, was first reported in 2015 [97]. Migrasomes are approximately 500 nm to 3 μm-sized membrane-bound structures and form at the tips or intersections of retraction fibres left behind by migrating cells, where they facilitate the transfer of cytosolic material to neighbouring cells [97]. In BC, migrasomes released by brain-tropic cancer cells transferred activating transcription factor 6 (ATF6) to brain endothelial cells, triggering endoplasmic reticulum stress, disrupting the blood–brain barrier, and promoting brain metastasis [98]. However, the commonly used isolation protocol for migrasomes, centrifugation at 20,000 × g for 30 min, is most likely to co-isolate a substantial proportion of lEVs. To date, there is no established method to robustly isolate and distinguish migrasomes from lEVs during isolation. In addition, migrasome characterisation largely relies on particle size and proposed subtype-specific markers. Zhao et al. identified N-deacetylase and N-sulfotransferase 1 (NDST1), phosphatidylinositol glycan anchor biosynthesis class K (PIGK), carboxypeptidase Q (CPQ), and EGF domain-specific O-linked N-acetylglucosamine transferase (EOGT) as proteins enriched in migrasomes but rarely detected in sEVs, indicating distinct protein profiles between these vesicle populations [99]. However, no studies have systematically compared marker panels between migrasomes and lEVs, despite the substantial overlap in their size ranges. Transmission electron microscopy (TEM) is considered the gold standard for validating the identification of the unique, distinct morphology of migrasomes. In general, lEVs typically appear as relatively large, cup-shaped vesicles, whereas migrasomes exhibit pomegranate-like structures [99]. However, TEM typically captures only a small fraction of a sample; while it can confirm the presence of the target subtype, it cannot reliably exclude co-isolation or contamination by other EV subtypes in the same preparation. Therefore, functional effects attributed to migrasomes, especially those related to metastasis, may not be exclusively mediated by migrasomes but could also involve co-isolated lEVs. As already discussed, lEVs themselves may also be significantly associated with BC metastasis. As migrasomes cannot yet be reliably distinguished from the broader lEV population, it remains difficult to conclude that migrasomes are the primary drivers of BC metastasis. Overall, the current data more strongly support a central role for lEVs in promoting BC metastasis. Collectively, a comprehensive understanding of metastasis requires moving beyond the study of isolated vesicle subtypes and focusing on the synergistic interactions between sEVs and lEVs.

### EV-based therapeutics for BC metastasis

EV-targeted and EV-based therapeutic strategies for BC metastasis include the inhibition of EV secretion, uptake, and trafficking, the modulation of EV cargo, and the use of engineered EVs as drug delivery vehicles, as summarised in Table 4. Inhibiting sEV secretion and uptake has emerged as a promising therapeutic strategy to prevent BC metastasis. Sulfisoxazole (SFX), an FDA-approved antibiotic, inhibited sEV biogenesis and secretion by disrupting endothelin receptor A expression [100]. This inhibition significantly reduced BC progression and metastasis in the mouse TNBC xenograft models. Temsirolimus, another FDA-approved anti-cancer drug, inhibited mTOR phosphorylation and activated autophagy, suppressing various tumour growth [101]. Park et al. reported that temsirolimus enhanced CD8^+^ T cell-mediated anti-cancer effects by inhibiting sEV PD-L1 secretion in TNBC cells [102]. Also, combining temsirolimus with anti-PD-L1 therapy increased CD4^+^ and CD8^+^ T cell activity, further suppressed sEV PD-L1 secretion, and accelerated anti-cancer immunity, thereby amplifying therapeutic efficacy. However, a Phase II clinical trial showed that temsirolimus monotherapy in BC has demonstrated limited activity in patients with heavily pretreated metastatic BC [103]. As this study did not evaluate TNBC or sEV PD-L1 inhibition, its therapeutic value in BC remains unclear and warrants further investigation.

**Table 4:** Therapeutic approaches targeting EV-mediated BC metastasis

Treatment/approach	EV-related mechanism	Main finding	Study model	Reference
Sulfisoxazole	sEV secretion	Reduced tumour growth and metastasis	*In vivo*	[100]
Temsirolimus + anti-PD-L1	Autophagy-mediated reduction of sEV PD-L1	Enhanced antitumour immunity preclinically; but limited clinical activity	*In vivo*	[102]
DisBa-01	sEV integrin-mediated uptake	Reduced sEV adhesion to fibronectin, sEV-related cell adhesion, and sEV uptake	*In vitro*	[104]
Trametinib	Inhibition of MEK2-dependent macropinocytosis	Reduced EV uptake by lung fibroblasts	*In vitro*	[105]
DMA + SB431542	Inhibition of sEV secretion and TGF-β signalling	Reduced tumour progression and metastasis	*In vivo*	[107]
IL-3Rα blockade	Reprogramming of tumour-endothelial-cell EV cargo	Reduced migration and distant metastasis Increased apoptosis	*In vivo*	[108]
miR-1-3p-enriched CAF EVs	GLIS1 suppression	Reduced tumour progression and metastasis	*In vivo*	[109]
Lactadherin immunoblockade	sEV lactadherin	Reduced ascites and tumour nodules	*In vivo*	[110]
HA/CV-conditioned, DOX-loaded M1 EVs	Drug delivery and TAM modulation	Reduced primary tumour growth and metastases	*In vivo*	[113]

Beyond suppressing vesicle release, inhibiting sEV uptake may also reduce metastatic progression. In TNBC cell lines, DisBa-01-mediated blockade of αvβ3 integrin reduced sEV binding to fibronectin, sEV-supported tumour-cell adhesion, and sEV uptake by non-malignant breast epithelial cells [104]. These findings suggest targeting sEV αvβ3 integrin may disrupt EV-mediated processes implicated in metastatic progression. Consistently, trametinib (a MEK1/2 inhibitor) inhibited uptake of TNBC-derived sEVs by lung fibroblasts, suggesting that it may reduce BC metastasis by blocking MEK2-dependent EV uptake [105].

Since TGF-β signalling plays a pivotal role in BC metastasis, targeting this pathway has also been explored, although clinical translation of TGF-β inhibitors remains challenging [106]. Teixeira et al. demonstrated that combining dimethyl amiloride (DMA) with SB431542 to inhibit sEV trafficking and TGF-β signalling, significantly reduced BC progression and metastasis *in vivo* by normalising TGF-β signalling levels [107]. Additional cargo-focused approaches have leveraged tumour-specific targets and reprogrammed vesicle contents. Interleukin-3 receptor alpha (IL-3Rα) was highly expressed in TNBC cells and anti-IL-3R-sEV was generated by treating tumour-endothelial cells with an IL-3Rα blockade [108]. These sEVs downregulated miR-24-3p, reducing liver and lung metastases by decreasing cell migration and promoting apoptosis *in vivo*. In the tumour microenvironment, sEV miR-1-3p was consistently downregulated in BC tissues and CAF-derived EVs compared with normal fibroblast-derived EVs [109]. Delivery of miR-1-3p-enriched CAF-derived EVs suppressed BC cell viability, invasion, migration, tumour formation, and metastasis by downregulating GLIS family zinc finger 1 (GLIS1) [109]. Lactadherin was enriched in sEVs derived from metastatic TNBC cells [110]. Blocking sEV lactadherin in mouse TNBC tumours reduced ascites and tumour micronodule formation, supporting its potential as a therapeutic target to limit tumour dissemination and metastasis.

Engineered sEVs have been explored as drug delivery vesicles to modulate the immune microenvironment. Jorquera-Cordero et al. generated sEVs from M1 macrophages pre-treated with hyaluronic acid (HA) and the β-blocker carvedilol (CV), based on evidence that HA-based formulations promote anti-tumour macrophage polarisation [111] and suppress β2-adrenergic signalling associated with M2 macrophage polarisation [112, 113]. Doxorubicin-loaded sEVs administered in a BC mouse model reduced tumour size and metastasis, suppressed NF-κB signalling-related genes, increased apoptosis, and decreased TAM abundance [113].

In summary, targeting EVs offers a promising therapeutic avenue for addressing BC metastasis. Strategies that inhibit EV release or uptake, interfere with EV trafficking, and therapeutically modulate EV cargo have shown substantial potential to limit tumour progression and metastatic spread. In addition, EV-based delivery platforms may enhance anti-tumour immunity and reshape the tumour microenvironment. However, because research on EV subpopulations beyond sEVs remains limited, translating EV-targeted strategies to manage heterogeneous and complex BC in clinical settings remains challenging. Accordingly, shifting towards analysis of cargo at the single-EV level, rather than relying solely on bulk EV profiling, will be critical for advancing mechanistic understanding and therapeutic development in BC.

### Non-vesicular EPs: exomeres and supermeres

Apart from EVs, several studies have identified non-vesicular EPs, such as exomeres and supermeres, with approximate diameters of 35 nm and 22–32 nm, respectively [114, 115]. Studies have also reported distinct organ distribution among these particles and EVs. In a mouse biodistribution study, exomeres and sEV subpopulations were predominantly taken up by the liver, whereas a larger sEV subpopulation showed additional lymph node tropism [114]. Supermeres showed high uptake by the spleen, liver, and kidney and, in particular, greater uptake in the brain than exomeres and sEVs [115]. Exomeres and supermeres also differ from EVs in their nucleic acid, protein, and lipid composition, which may result in distinct functions in cancer [116]. Proteomic profiling of exomeres revealed an enrichment in metabolic enzymes, glycolysis and mTOR signalling pathways, whereas the exosome subpopulations were enriched in proteins of endosomal function and secretion [114]. Supermeres are morphologically distinct from exomeres and contain cargo associated with several types of cancer [115]. Although earlier studies often detected extracellular miRNAs in sEVs or lEVs using conventional EV isolation methods, newer separation methods have shown that extracellular RNAs are also present in EPs, particularly supermeres [115, 117]. This suggests that some miRNAs previously described as sEV miRNAs may also be enriched in amembranous EPs, and that the composition and functional attribution remain largely unexplored [118]. In BC, tumour cells have been reported to release extracellular ST6 beta-galactoside alpha-2,6-sialyltransferase 1 (ST6GAL1) in association with sEV-like and exomere-like particles; however, the specific functional contribution of purified exomeres remains unclear [119]. This cargo distribution directly affects how we should interpret the metabolic mechanisms described previously. The metabolic remodelling attributed to sEVs during pre-metastatic niche formation and organ colonisation is precisely the effect for which glycolytic enzymes and metabolic-pathway proteins are most enriched in the non-vesicular fractions. Because conventional differential ultracentrifugation and precipitation methods do not separate exomeres and supermeres from sEVs, a contribution from co-isolated non-vesicular particles cannot be excluded. We therefore reasonably regard the mechanisms summarised in Tables 1 and 2 as attributable to sEV-containing preparations rather than to sEVs as a purified entity, and the same caution should apply to the lipid-composition differences discussed above. Moreover, as a result of the co-isolation, an effect observed with a bulk pellet should be described as EV-associated rather than EV-mediated. This ambiguity between association and genuine transfer remains one of the principal unresolved challenges in the field [120].

Beyond their potential biological roles, the heterogeneity of EVs and non-vesicular EPs may also be relevant to biomarker discovery. Hoshino et al. found that proteomic profiles of tissue- and plasma-derived EVs and EPs could distinguish tumour from normal samples and help identify the tumour of origin [121]. These findings support the use of a broader range of EV- and EP-associated proteins in biomarker panels. Further studies are needed to clarify the relationship between EPs and EVs and to determine their specific roles in BC. Reflecting this, an inclusive term, extracellular vesicles and particles (EVPs) has been adopted recently to describe the population as a whole, without committing to a defined vesicle/particle class [122]. Nevertheless, these findings suggest that bulk EV preparations may include several biologically distinct particle populations, underscoring the importance of subpopulation-resolved and single-particle analyses.

## Single-EV analysis for BC diagnosis and prognosis

**Fig. 2:**
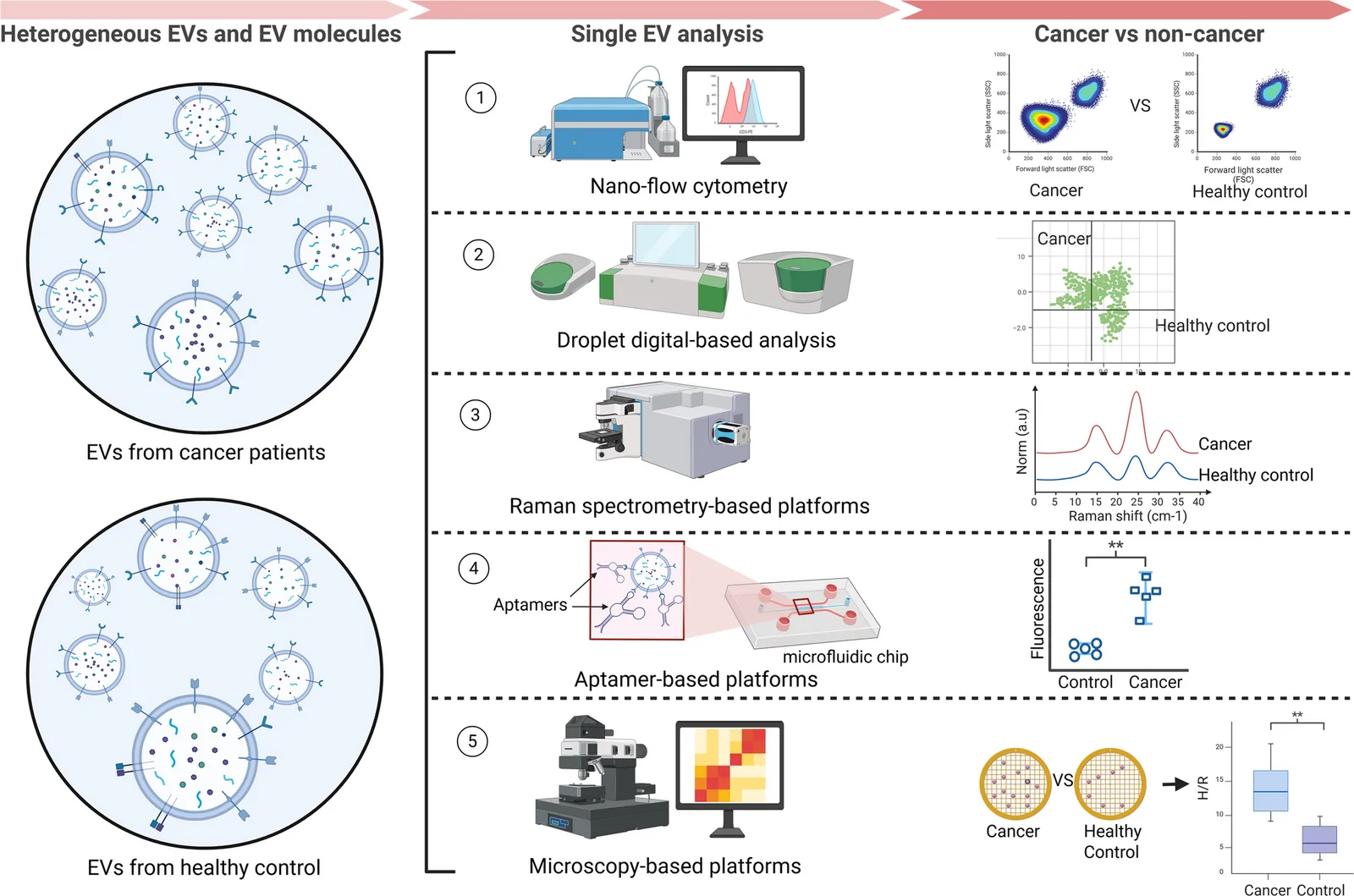
Single-EV analysis enables the detection of heterogeneous EV populations from cancer and non-cancer sources. EVs are heterogeneous in size and molecular composition, even when derived from the same conditioned sample. In addition, specific EV cargoes present within EVs or on their surface are often low in abundance. Therefore, single-EV analysis is needed to characterise individual EVs in detail beyond bulk profiling. In this review, single-EV analysis methods are broadly grouped into five categories: nano-flow cytometry-based, droplet digital-based, Raman spectrometry-based, aptamer-based, and microscopy-based platforms. This figure shows how these approaches differentiate and analyse cancer-associated EV subtypes relative to non-cancer controls in cell-derived and clinical samples. Created in BioRender. BioRender.com/wili8t2

It has been well established that EVs are heterogeneous in size, surface characteristics and cargo composition [123]. Since EV cargo determines its biological functions in cancer, precise characterisation of EV subpopulations is crucial [10]. However, identifying tumour-derived EVs within the large background of host-derived EVs is highly challenging, as heterogeneity is often driven by only a subset of cells within the total population, such as tumour cells [124]. Furthermore, given that the size of EVs varies depending on the subpopulations, ranging from 30 to 1000 nm, the application of advanced analytic technology requires accurate particle characterisation. To address these challenges, single-EV analysis has gained attention for detecting EVs carrying specific surface biomarkers [125]. By moving beyond bulk measurements which average molecular signals and mask subpopulation heterogeneity, single-vesicle technologies enable the simultaneous measurement of multiple biomarkers within individual vesicles [126], aiding in cancer diagnosis and prognosis through high-resolution profiling of single EVs. A recent review has broadly categorised single-EV technologies into optical and mechanical characterisation methods, as well as multiplexed profiling platforms using substrate-, droplet- and solution-based strategies [127]. In these platforms, individual EVs are analysed on substrates, within droplets, or directly in solution. Fluorescence or molecular coding can be used to detect multiple markers although each approach differs in sensitivity, throughput, and multiplexing capacity. Against this background, this section examines representative current single-EV analysis techniques, as in Fig. 2 and their diagnostic and prognostic implications in BC, as summarised in Table 5.

**Table 5:** Summary of current single-EV analysis platforms

Single-EV methods	Target/readout	Functional relevance	Strengths	Limitations	Applications	Clinical translation potential
nFCM	Light scattering and fluorescence of single-EV surface markers	Define membrane heterogeneity at the single-EV level, which identifies EV subpopulations	High sensitivity, multiparametric phenotyping, and subpopulation profiling	Antibody specificity, platform dependence, spectral overlap, and limited multiplexing	Biomarker discovery and EV subpopulation analysis	EV size and surface protein biomarker profiling in colorectal cancer patient samples [131]
Droplet digital-based platforms	Absolute digital quantification of EV surface proteins and nucleic acids	Enables sensitive profiling of low-abundance EV cargo and heterogeneity	High sensitivity and specificity, absolute quantification, and high signal-to-noise performance	Pre-analytical variability, limited multiplexing, and specialised equipment required, droplet instability	Targeted diagnosis and treatment monitoring	Multiplexed sEV mRNA profiling using plasma samples from a clinical BC cohort supported molecular subtype classification and diagnostic model development [142]
Raman spectrometry based platforms	Label-free or labelled spectral profiling of single-EV proteins and nucleic acids	Captures compositional heterogeneity of EVs and tumour-associated molecules, enabling discrimination between cancer and non-cancer	Provides multiplex molecular fingerprints and label-free classification	Spectral overlap, signal distortion, Raman peaks ambiguity to specific EV biomarkers, low reproducibility, and limited clinical validation	Exploratory biomarker discovery and molecular classification	Evaluated serum-sEV profiling for early detection, staging and subtyping of lung cancer [183]
Aptamer-based platforms	Selective detection and capture of surface protein of single EVs	Reveals tumour associated surface molecules and heterogeneity across EV subsets	Target specificity, stability, and strong adaptability for selective EVs and heterogeneous EV populations	Clinical translation required further validation	Targeted EV detection	Applied to the detection of MUC1-positive sEVs in BC plasma samples [184]
Microscopy-based platforms	Biophysical properties, biological molecules of EVs signals, and uptake or trafficking of single EVs	Captures mechanical heterogeneity, nanoscale molecular phenotypes, and EV interaction dynamics related to cancer phenotypes	Direct single-EV visualisation, high-resolution analysis of structure, mechanics, and molecular contents, and supports functional interrogation	Low throughput, highly laborious, special instruments required, and limited suitability for clinical application	Mechanistic and structural studies	Direct plasma EV profiling for early detection and discrimination of HER2^+^ and HER2^−^ BC [181]

### Nano-flow cytometry for single-EV analysis

Conventional flow cytometry (FCM) typically has insufficient sensitivity and resolution to reliably detect particles smaller than 500 nm, making it inadequate for EV analysis [128]. To overcome this gap, researchers have developed highly sensitive nano-flow cytometry (nFCM) [129–131]. This technique enables the detection of single-particle light scattering from viruses, silica nanoparticles, and gold nanoparticles as small as 30 nm [132]. Additionally, nFCM allows for multiparameter fluorescence, allowing protein profiling of single EVs as small as 40 nm [131]. A laboratory-built high-sensitivity nano-flow cytometry system with a two-channel setup has been developed for the multiparameter detection of surface-labelled EVs using two-colour fluorescence [133]. Recent studies have utilised nFCM for single-EV analysis, to quantify specific EVs by detecting target proteins on their surface, as well as to characterise various EV subpopulations using single-particle light-scattering detection with multiparameter fluorescence [130]. One study successfully applied nFCM to simultaneously distinguish lEVs and sEVs by detecting the commonly used sEV marker CD63 and the lEV marker annexin A1 [134]. The results revealed a significantly higher level of lEVs in BC cell lines compared to non-cancerous cells. Furthermore, the simultaneous detection of annexin A1 or CD63 with annexin A5, which is a protein binding to phosphatidylserine on the surface of EVs, demonstrated that lEVs derived from BC cell lines exhibited higher phosphatidylserine levels than sEVs. This suggested that phosphatidylserine of lEVs might have a greater potential for use as a BC diagnosis marker than sEVs. Additionally, glypican-1^+^ sEVs were identified from BC patients' plasma samples using nFCM, showing elevated glypican-1^+^ sEVs which increased with BC stage [135]. This suggested that sEV glypican-1 could serve as a potential prognostic marker for BC staging.

Despite these advancements, nFCM is not without limitations. Common drawbacks include reduced detection sensitivity due to limited antibody specificity for EV surface epitopes, dispersion of emitted light across the channels, and spectral overlap between fluorophores [136]. In addition, the reliability of single-EV detection remains highly platform-dependent. Kim et al. reported that, when comparing commercial high-sensitivity flow cytometers and a custom single-molecule flow cytometer, both marker fluorescence detection limits and size detection limits differ substantially, leading to large discrepancies in the measured proportions of EV subpopulation [137]. These findings highlight the need for further optimisation and standardisation to support clinical translation. Sample dilution is another important source of error in EV flow cytometry. Rops et al. described a “dilution paradox”, in which the dilution required to prevent swarm detection may be so high that labelled EVs can no longer be reliably distinguished from fluorescent background signals [138]. Together, these limitations make it challenging to achieve the sensitivity and specificity required for the clinical application of nFCM in cancer diagnosis and prognosis. This challenge is further complicated by variability in EV isolation methods. Currently, EV isolation techniques are not standardised, leading to significant variation among findings [8]. If specific surface proteins on EVs can be reliably detected from a small blood volume without requiring isolation, this would not only standardise results but also streamline workflow, improving speed, reproducibility, and consistency in clinical laboratory settings. Furthermore, since multiple EV protein sets have been identified as highly sensitive and specific biomarker panels for cancer detection [139], increasing the number of simultaneously detectable proteins could enhance the accuracy of EV-based diagnostics. Therefore, expanding multiplexing capabilities beyond two detection channels is crucial for enhancing clinical applicability.

### Droplet digital based single-EV analysis

Droplet digital PCR (ddPCR) offers greater sensitivity than conventional PCR and enables precise absolute quantification of target nucleic acids [140]. In BC, EV microfluidic affinity purification combined with ddPCR enabled the analysis of epithelial cell adhesion molecule (EpCAM)^+^ and fibroblast activation protein alpha (FAPα)^+^ sEV-associated transcripts for molecular subtype classification [141]. To further increase throughput, Liu et al. developed a 4-plex ddPCR to detect four different sEV mRNAs, such as PGR, ESR1, ERBB2, and GAPDH simultaneously in BC plasma samples [142]. The data were analysed using machine learning algorithms to construct diagnostic models and evaluate the efficacy of four different sEV mRNA combinations, demonstrating an optimal combination of biomarkers for BC diagnosis. Most recently, they also constructed droplet digital immuno-PCR (ddiPCR), which combined ddPCR and immuno-PCR [143]. Importantly, ddiPCR can concurrently profile surface proteins of individual sEVs, including CD9/CD63/CD81, human epidermal growth factor receptor 2 (HER2), and EpCAM in BC plasma samples. This demonstrated a diagnostic value with 91.30% sensitivity and 90.01% specificity. Mugoni et al. employed ddPCR to identify higher levels of sEV *ERBB2* RNA and circulating sEV *ERBB2* DNA in HER2^+^ early BC patients’ plasma samples compared to healthy donors [144]. Their findings highlighted the highly sensitive biomarker potential of ERBB2/HER2 in early BC detection. However, ddPCR has limitations, including the lack of standardised methods for sample preparation and the requirement for specialised equipment, which restricts accessibility [145]. Conventional western blotting has been used to validate the relative quantification of proteins of EVs. However, its results can be affected by contaminants in EV samples and do not provide absolute protein quantification. To address this limitation, a droplet digital single-exosome-counting enzyme-linked immunoassay (ddExoELISA) was developed by using antibody-coated and enzymatic tagged-magnetic beads to capture single sEVs [146]. The captured beads are encapsulated within microdroplets, and during the catalysis of tagged enzymes, the emitted fluorescence is detected to calculate the concentration of sEVs. In this study, ddExoELISA found overexpressed Glypican-1 (GPC-1)^+^ sEVs in BC patient serum samples when compared to both non-cancer controls and post-surgery samples, suggesting that the detection of GPC-1^+^ sEVs by ddExoELISA may be a suitable method for diagnosing BC and monitoring postsurgical outcomes. The study reported that ddExoELISA can quantify cancer-specific EVs with a detection limit as low as 10 enzyme-labelled EVs per microlitre.

The digital platforms represent a substantive advance in signal-to-noise performance for EV detection and profiling [147]; however, significant barriers to routine clinical implementation remain. Technologies like ddPCR and ddExoELISA mitigate background interference by compartmentalising reactions, reaching high resolution but also rendering the data highly susceptible to pre-analytical variables, particularly when EV isolation and enrichment workflows remain non-standardised. Additionally, while digital quantification offers exceptional sensitivity, it often necessitates a trade-off in multiplexing capabilities, as droplet assays are often restricted by spectral limitations or binary enzymatic readouts (such as ddExoELISA) [148]. Beyond these analytical design limitations, droplet-based analyses also face challenges related to emulsion stability and droplet coalescence due to their reliance on oil and surfactant formulations [149]. Alternatively, Morasso et al. applied a droplet-free, microwell-based immunoassay, Single Molecule Detection Array (SiMoA), which can mitigate several practical limitations of the droplet-based assay [150]. Using this assay, they directly quantified CD9^+^/CD63^+^ sEVs in plasma without sEV isolation steps and reported higher CD9^+^/CD63^+^ sEV levels in BC patients when compared to healthy samples. However, SiMoA does not fully address pre-analytical variability in EV preparation due to lipoprotein and EV subpopulation bias, and multiplexing remains constrained by assay design and spectral overlap [151].

### Raman spectrometry based single-EV analysis

As previously reviewed in [152], Raman spectroscopy (RS) is a highly sensitive technique for analysing the chemical composition of EV cargo, based on detecting light scattering that reflects molecular vibrations when monochromatic light interacts with the sample. RS is typically divided into two categories, labelled and label-free RS [153]. Label-free is used for broad screening or profiling EVs through clustering and classification. One study integrated *nata de coco* as an alternative to bacterial cellulose membranes with an *in-situ* silver nanoparticle (AgNP) synthesis for label-free Surface-enhanced Raman spectroscopy (SERS) [154]. The principal component analysis successfully distinguished sEVs derived from TNBC cell lines compared to normal breast cell lines. However, label-free techniques for EV analysis pose challenges due to overlapping peaks and variations, making it difficult to classify EVs [155]. Another study utilised labelled SERS, a method for quantifying sEVs by measuring the scattering laser signal from sEVs treated with nanoparticles [156]. However its accuracy is compromised due to signal distortion arising from the various sizes of EVs [157]. In response to this limitation, scientists have developed advanced techniques beyond SERS that allow for the detection of EVs on an individual basis. One such approach is single particle automated Raman trapping analysis (SPARTA), an advanced BC diagnostic system based on RS with optical trapping. SPARTA successfully distinguished BC cell-derived single sEVs from non-cancerous cell-derived single sEVs [158]. By providing a comprehensive fingerprint of sEV composition, such as nucleic acids and proteins, SPARTA enables the identification of distinct spectral differences in single sEVs. Using this approach, sEVs derived from BC and non-cancer breast cell lines could be distinguished with over 95% sensitivity and specificity, while further modelling also enabled accurate classification of EVs from closely related BC subtypes [158]. Therefore, SPARTA holds promise for cancer/non-cancer classification at the biomolecular level. Nevertheless, its application is constrained by the requirement of a higher volume of sEVs and the necessity of further investigation for its transition to clinical usage. Recently, Wang et al. [159] developed microdroplet-based SERS (microdroplet SERS) to detect sEV proteins using an in-drop immunoassay to differentiate between heterogeneous BC subtypes. sEV proteins were bio-conjugated with immuno-SERS tags containing sEV markers CD9, CD63, and focal adhesion kinase (FAK). This conjugation amplified the signals for SERS detection, revealing significantly different expression levels of FAK and heterogeneous distribution of CD63 and CD9 between BC cell lines MDA-MB-231 and MCF7. The authors suggested that the detected heterogeneous distribution of sEV markers via SERS can be used for metabolic analysis or drug analysis at a single-cell level in the future. Although RS has significantly advanced, it remains limited by the challenges in identifying suitable biomarkers and Raman tags for EVs, as well as establishing a clear relationship between specific Raman peaks and EV biomarkers [152, 160]. Additionally, complex labelling steps, lengthy processes, and low reproducibility due to alterations in EV properties hinder the practical application.

Recently, artificial intelligence (AI) technology has gained tremendous attention from researchers. In particular, convolutional neural networks (CNNs) have been used to analyse complex Raman spectra of bacteria, successfully distinguishing bacterial strains with an accuracy of 95–100% by detecting unique spectral patterns [161]. These techniques have now been effectively translated to EV analysis; for instance, an AI model trained on EV data from lung cancer cell lines was able to classify lung cancer patients with 90.7% accuracy [162]. By integrating large datasets of Raman peaks and EV molecules, AI can facilitate the accurate differentiation of cancer-related EVs from background noise. Moreover, even without specific labelling, cancer diagnosis can be achieved by identifying unique spectral signatures of cancer-related EVs, eliminating the need for complex labelling procedures. However, two major challenges remain: first, collecting large-scale human datasets is difficult because human EVs are highly heterogeneous and complex; second, the massive data size makes validation challenging, affecting data reliability. Despite these hurdles, AI holds great promise for advancing EV biomarker research across various diseases.

### Aptamer-based single-EV analysis

Aptamers, often referred to as “chemical antibodies”, are short nucleic acid sequences (oligonucleotides) that bind to specific target proteins with high affinity and specificity [163]. Due to their small size, stability, and modifiability, aptamers can be easily engineered or designed for capturing heterogeneous single EVs and distinguishing cancer-derived EV signatures [163, 164]. Several studies have demonstrated aptamer-based isolation and detection methods for individual EVs. A DNA aptamer specifically detected HER2 and EpCAM on the surface of single EVs, and increased expression of HER2 and EpCAM was shown in BC cells and plasma samples [165, 166]. Liu et al. introduced additional separation steps after the aptamer-based isolation using λ-DNA-mediated viscoelastic microfluidics to distinguish EV subpopulations such as sEVs, lEVs, and apoptotic bodies based on their size [165]. A subsequent application of a machine learning algorithm demonstrated that elevated expression of HER2 and EpCAM on lEVs in BC patients, compared with healthy controls, provided greater discrimination power relative to their expression on other EV subpopulations. In another study, a DNA aptamer-based approach was used to detect sEVs in plasma for BC diagnosis [167]. Two single-stranded DNA aptamers were designed to bind the cancer marker mucin 1 (MUC1) on sEV surfaces and a fluorescent dye, respectively. These aptamers were assembled with gold nanoparticles and micro-structured fibres, enabling detection of increased levels of MUC1^+^ sEVs in BC plasma samples compared to healthy controls. Collectively, aptamer-based strategies represent a promising direction for improving BC diagnostics, offering high adaptability and sensitivity beyond that of conventional antibody-based methods. However, despite strong performance in controlled experimental settings, further studies are needed to validate their reliability and robustness in complex clinical samples.

### Microscopy-based single-EV analysis

Microscopy-based techniques are increasingly playing a crucial role in single-EV detection. These approaches can be broadly categorised as label-free and labelled technologies. Label-free microscopy techniques allow for the analysis of EVs without tags or dyes, offering essential insights into their biophysical and structural properties [168], which include atomic force microscopy (AFM), near-field infrared spectrometry, and plasmonic imaging. Ye et al. developed an intriguing method applying AFM to analyse sEV stiffness through measuring bending modulus and osmotic pressure, by analysing the deformed radius of the sEVs caused by tip indentation and the actual height of the sEVs without tip indentation [169]. This study demonstrated how sEV stiffness can serve as a diagnostic indicator to assess BC malignancy [169]. AFM combined with scanning ion conductance microscopy (SICM) was applied to analyse the dynamics and interactions of sEVs at the single vesicle level by assessing their mechanical response [170]. The stiffness calculated from TNBC cell line sEVs was found to be significantly increased compared to normal breast cells, correlating with the malignancy of the BC cells. This ability to distinguish highly malignant BC-derived sEVs from those of normal controls highlighted the diagnostic potential of this combined technique, offering high sensitivity and specificity. Another single-EV analysis method is near-field infrared (nano-FTIR) spectrometry, which combines infrared microscopy and AFM with a spatial resolution of 20 nm [171]. A study that utilised nano-FTIR spectrometry to gauge the optical absorption of molecules, assessed the heterogeneity of sEVs’ secondary structures, suggesting these factors could be used as potential biomarkers of tumour malignancy in BC [172]. In particular, a rise in the amide I/II absorption ratio of sEVs and the proportion of β-sheet and β-turn in sEV proteins were found to be positively related to BC malignancy. Conversely, the proportion of α-helix and random coil in sEV proteins demonstrated a negative association with BC malignancy. Overall, the study concluded that the spatial heterogeneity and absorption of sEV proteins effectively distinguish metastatic BC with high specificity and sensitivity. In addition to AFM- and IR-based modalities, plasmonic imaging emerged as a label-free strategy for single-EV detection. Daraei et al. developed a microfluidic chip integrated with an arrayed gold nanostructured sensor surface (AGNIS) that enables direct capture of plasma sEVs via canonical EV markers CD63, CD9, and CD81 without the need for prior purification [173]. Single-EV binding events were detected using a label-free localised surface plasmon resonance (LSPR)-based imaging platform, with the assay revealing a higher abundance of sEVs in BC plasma compared to healthy controls.

Labelled techniques, by contrast, rely on labelling and affinity strategies to detect, quantify, and characterise limited selected single-EV populations and their molecular cargoes [168]. Labelled microscopy-based methods can be further divided into conventional fluorescence imaging, super-resolution microscopy, single-particle tracking, and hybrid imaging approaches. Total internal reflection fluorescence (TIRF)-based single-vesicle imaging assay is a widely used technique for visualising and quantifying single EVs and their molecular cargoes [174]. For example, one recent study used DNAzyme-based fluorescent probes targeting miRNAs within sEVs followed by a TIRF assay, and reported a reduced sEV miR-21 level in serum samples from BC patients post chemotherapy [175]. Another study introduced microfluidic-chip based EV capture for TIRF detection [176]. Here, a single cell was isolated in a microchamber, and secreted sEVs were captured and immunostained on-chip, enabling fluorescence detection of individual sEVs by TIRF microscopy. Using HER2 and estrogen receptor alpha (ERα) as markers, the study detected HER2^+^ and ER^+^ sEVs and differentiated BC cell lines with distinct molecular phenotypes. Single-molecule localisation microscopy (SMLM), a super-resolution imaging technique, enables precise spatial quantification of individual EVs by detecting EV marker antibodies conjugated with fluorophores [177]. Jiang et al. combined affinity-based sEV isolation with SMLM to enhance the precision of single sEV detection and profiling of HER2-enriched sEVs from BC cell lines and BC patients’ plasma [178]. Another SMLM approach, direct stochastic optical reconstruction microscopy (dSTORM), was used by Wilhelm et al. to visualise CD9 and HER2 on single EVs derived from BC cell lines [179]. This analysis confirmed the co-expression of CD9 and HER2 on EVs released from HER2^+^ BC cell lines, but not on EVs from HER2^−^ ones. Single-particle tracking assay can be applied to track the transfer or uptake of DiO and DiL dye-labelled single sEVs using a fluorescence microscope in real time [180]. For example, when tracking the migration trajectory of tetraspanin 8 (TSPAN8) enriched-single sEV on the BC cell membrane surface, a significant confined diffusion was observed, suggesting TSPAN8 might facilitate the binding and docking of sEVs to the target cells [180]. Finally, several hybrid imaging strategies combine scattering and fluorescence-based readouts to improve tumour EV discrimination. The superficial dual imaging single vesicle technology (DISVT) differentiated tumour-derived sEVs by detecting light scattering from gold nanoparticle binding to the target surface proteins of sEVs and fluorescence [181]. Using DISVT, plasma samples from one study differentiated HER2^+^ BC patients and identified a positive correlation between the clinical stage of HER2^+^ BC and quantified HER2^+^ sEVs. Another study, rolling circle amplification with expansion microscopy (RCA-ExM), was used to analyse EV membrane proteins and miRNA phenotypes at the single-EV level using hairpin probes [182]. Utilising EpCAM^+^/PD-L1^+^ EVs from plasma across various cancer types, the study observed increased sEV PD-L1 signals after treatment and assessed changes in sEV miR-21 levels. However, the post-treatment analysis was performed in only a limited subgroup of patients with paired pre- and post-treatment samples, which restricts the generalisability of these findings and highlights the need for validation in larger longitudinal cohorts.

Although single-EV studies in BC have focused mainly on diagnosis and prognosis rather than metastasis, emerging evidence suggests that these approaches may also provide insight into metastatic disease. Nano-FTIR analysis showed that the structural characteristics of proteins within individual sEVs could distinguish metastatic BC [172]. Similarly, single-particle tracking revealed distinct binding and uptake behaviours of TSPAN8-enriched sEVs, which may be relevant to EV-mediated metastatic processes [180]. Although evidence remains limited, single-EV profiling could help identify metastasis-associated EV surface molecules and molecular cargo, providing a basis for the discovery of potential therapeutic targets.

## Conclusion and future perspectives

This review highlights that EV subpopulations, including sEVs and lEVs, actively contribute to BC metastasis. RNAs and proteins carried by sEVs and lEVs have been extensively investigated for their roles in metastatic progression, suggesting that EV cargoes may serve as potential therapeutic targets. Specific RNAs and proteins within EVs can remodel the TME through immune modulation, stromal reprogramming, and inflammatory signalling during both pre-metastatic and pro-metastatic stages, including organotropism in BC. A clearer understanding of the cargo composition and functional properties of each EV subpopulation is, therefore, necessary to elucidate metastatic biology and identify actionable therapeutic opportunities.

However, the marked heterogeneity of EVs underscores the need for single-EV analysis to capture the spectrum of functionally relevant particles. EVs vary widely in size, surface markers, and cargo composition, while tumour-derived EVs are typically rare within the broader circulating EV population. As a result, single-EV analysis enables the interrogation of individual vesicles and the multiplex detection of co-localised biomarkers on the same particle, thereby improving the accuracy of cancer diagnosis and prognosis. Accordingly, this review discusses recent advances in single-EV technologies and summarises their application in BC research. By linking EV biology with single-particle analytical platforms, this approach provides a framework for connecting EV function with clinically measurable biomarkers.

Despite recent progress, the clinical translation of single-EV analysis remains limited by several challenges. A major limitation is the extremely low abundance of tumour-derived EVs, which are estimated to comprise as little as 0.0001% of total EVs in human plasma [185]. For instance, mRNA within sEVs derived from lung cancer, hepatocellular carcinoma, and multiple myeloma is both scarce and unevenly distributed [186], while glioblastoma-derived EVs may contain fewer than one copy of commonly mutated RNA per EV [187]. This challenge is further compounded by the low copy number and stochastic distribution of EV cargo. Proteomic studies similarly indicate that only 0.02 to 0.05% of proteins in plasma sEVs originate from BC [124]. Mathematical modelling suggests detecting EVs released by small tumours may require at least 100 vesicles per millilitre, highlighting the need for highly sensitive detection methods with minimal background interference [188].

Conventional bulk analysis often dilutes these rare tumour-derived signals into background noise, resulting in poor reproducibility and limited sensitivity. This limitation is particularly relevant for early detection and measurable residual disease monitoring, which are key clinical applications of EV-based liquid biopsies. To address this issue, Li et al. developed NanOstirBar-enabled Single Particle Analysis (NOBEL-SPA), a superparamagnetic nanorod-based tool capable of rapidly capturing EVs under a rotating magnetic field while simultaneously detecting protein and miRNA markers on single EVs [189]. Using this approach, the authors detected co-localised sEV proteins CD63 and miR-122 in serum and distinguished stage I and stage II BC patients from healthy controls using fewer than 100 EVs per μL of serum. Although this platform showed strong analytical performance, further validation across diverse clinical sample sets is required before it can be adopted in routine clinical practice.

Second, the field faces upstream bottlenecks, including the lack of standardised EV isolation, purification, storage, and normalisation protocols and the absence of universal identification markers. Current isolation strategies involve an inherent trade-off between purity and yield, and may enrich different EV subpopulation or co-isolate non-vesicular material, contributing to variability across studies [190, 191]. Moreover, storage temperature, storage duration, and repeated freeze–thaw cycles can affect EV concentration, structural integrity, marker availability, and cargo stability [192, 193]. Importantly, the choice of isolation method can alter downstream molecular readouts, including proteomic and transcriptomic profiles [194, 195]; so method variation is not merely noise but can change the biological conclusion. The MISEV guidelines therefore recommended the need for detailed reporting of pre-analytical and analytical procedures to improve reproducibility and inter-laboratory comparison [8]. In addition, the “universal” EV surface markers used in bulk analysis, such as CD9, CD63, and CD81, are not evenly distributed across all vesicles within a single sample [196]., This issue reflects the broader challenge of marker specificity and sensitivity. In single-EV analysis, at least one marker is required to confirm EV identity independently of the target cancer biomarker; however, relying on a single marker potentially leads to the exclusion of relevant EV subpopulations, while multiplexing markers consumes valuable detection channels (such as in nano-FCM) needed for cancer-specific biomarkers. Furthermore, depending on marker sensitivity and antibody performance, assay efficiency and reproducibility may vary. Using multiplexed universal EV markers may help address these limitations [197], but due to technical constraints such as limited sensitivity, low capture efficiency, and accessibility issues, this approach is not yet widely feasible. This limitation becomes particularly relevant because surface markers are often used not only to confirm EV identity but also to detect and quantify cancer-associated biomarkers and to define EV subtypes. Many EV-associated proteins can reside in both surface-exposed and intravesicular compartments [198]. Therefore, single-EV detection restricted to surface-accessible cargoes may not fully reflect total biomarker abundance in cancer. Reliance on surface profiling alone may provide an incomplete view of EV biology. Future efforts should therefore move toward single-EV multi-omics strategies that integrate surface marker profiling with intravesicular cargo analysis, allowing more comprehensive characterisation and accurate identification of cancer-associated EV subpopulations.

Thirdly, the high fabrication costs and limited availability of such advanced instruments continue to hinder the widespread adoption of single-EV technologies. To address this problem, some researchers have attempted to adapt existing research or clinical infrastructure. One example is “one-to-one fusion technology”, where sEVs are fused with a liposome to enlarge their size to over 1 μm, making them compatible with conventional flow cytometry [199]. Using this approach, elevated expression levels of four BC-associated proteins were detected in plasma sEVs from the BC patients compared to non-cancer controls. The method was reported to be highly cost-effective and suitable for rapid single-EV analysis without the need for specialised equipment. Nevertheless, its clinical utility remains uncertain due to the small patient cohort, and the selectivity and sensitivity for early-stage BC have yet to be firmly established. Further cost reduction and workflow simplification will be necessary to translate single-EV assays from specialised laboratories into routine clinical practice.

Finally, research on EV subpopulations beyond sEVs, lEVs, exomeres, and supermeres remains limited, and understanding the full functional landscape of EVs in cancer is still challenging. Further investigation of EV subtypes requires reliable discrimination between EV classes and the ability to analyse each at the single-particle level. As distinct protein and RNA profiles have been reported for EV subpopulations in BC [114, 115], each subtype may carry unique functions that are important for a complete understanding of BC biology and treatment response. Integrating single-EV profiling with functional validation will be essential to establish causal links between EV signature and metastatic phenotypes.

Despite these challenges, single-EV analysis remains highly promising because it enables direct investigation of intrinsic heterogeneity within EV populations. As technologies continue to improve in sensitivity, throughput, and accessibility, the resolution and reliability of single-EV profiling are expected to advance substantially. Therefore, continued development of single-EV analysis may hold strong potential to transform EV research and significantly enhance its clinical utility, particularly by uncovering previously unrecognised EV functions in cancer, as well as improving cancer diagnosis and treatment.

Because EV-based therapies are still in the early stages of clinical translation, continued advances in EV functional studies, isolation techniques, and characterisation methods will be crucial to overcome current challenges. Future studies should prioritise standardising EV preparation protocols and improving strategies for EV identification to enhance reproducibility and therapeutic efficacy. In addition, while most functional studies have focused on sEV miRNAs involved in BC metastasis, exploring the roles of lEV cargoes and sEV-associated proteins may uncover novel therapeutic strategies. Ultimately, a deeper understanding of EV biology will further support the development of EV-based diagnostic biomarkers and targeted treatments for BC, contributing to personalised and precision medicine. For clinical translation, single-EV biomarkers must be validated in multicentre, adequately powered cohorts using harmonised pre-analytical workflows and blinded study designs. Incorporating longitudinal sampling will also be essential to assess their performance in monitoring therapy response and detecting measurable residual disease. Given the rapidly expanding literature in this field, we acknowledge that this review may not capture every relevant study, and some important contributions may have been inadvertently omitted.

## Data Availability

No datasets were generated or analysed during the current study.
